# Listening in Pheromone Plumes: Disruption of Olfactory-Guided Mate Attraction in a Moth by a Bat-Like Ultrasound

**DOI:** 10.1673/031.007.5901

**Published:** 2007-11-26

**Authors:** Glenn P. Svenssona, Christer Löfstedt, Niels Skals

**Affiliations:** ^1^ Department of Ecology, Lund University, SE-223 62 Lund, Sweden. Center for Sound Communication, Institute of Biology, University of Southern Denmark, DK-5230 Odense M, Denmark

**Keywords:** *Plodia interpunctella*, sex pheromone, ultrasonic hearing, bat-moth interaction, Pyralidae

## Abstract

Nocturnal moths often use sex pheromones to find mates and ultrasonic hearing to evade echolocating bat predators. Male moths, when confronted with both pheromones and sound, thus have to trade off reproduction and predator avoidance depending on the relative strengths of the perceived conflicting stimuli. The ultrasonic hearing of *Plodia interpunctella* was investigated. A threshold curve for evasive reaction to ultrasound of tethered moths was established, and the frequency of best hearing was found to be between 40 and 70 kHz. Flight tunnel experiments were performed where males orienting in a sex pheromone plume were stimulated with 50 kHz pulses of different intensities. Pheromone-stimulated males showed increased defensive response with increased intensity of the sound stimulus, and the acoustic cue had long-lasting effects on their pheromone-mediated flight, revealing a cost associated with vital evasive behaviours.

## Introduction

Animals, which are exposed to a risk of predation, often alter their foraging or mating behaviour (Metcalfe et al. 1987; Forsgren 1992; Jones et al. 2002; Greenfield and Baker 2003). The final behavioural decision should reflect the relative strengths of conflicting sensory stimuli that they detect. Many species of nocturnal moths rely on chemical cues to find mates. Females release sex pheromones to attract males from large distance (Cardé and Baker 1984). Thus, a male moth may spend a considerable time in flight during a night searching for pheromone plumes, and as a consequence suffer a high risk of predation by aerially hawking bats (Acharya 1995). The strong selection pressure imposed by echolocating bats has triggered the evolution of ultrasonic hearing in several families of moths for detection and evasion of the predator (Roeder 1962; Fullard and Yack 1993; Miller and Surlykke 2001).

Extensive research has focused on the pheromone-mediated flight behaviour of moths (e.g. Baker and Vickers 1997; Cardé and Mafra-Neto 1997), as well as their evasive flight reactions in response to ultrasound (Roeder 1962; Rydell et al. 1997). Until recently, few studies had integrated these research fields. However, there are now accumulating data showing that ultrasound exposure affects the odour-guided flight behaviour of moths during their search for mates (Baker and Cardé 1978; Acharya and McNeil 1998; Skals et al. 2003a; Svensson et al. 2003; 2004), oviposition sites (Svensson et al. 2003), and nectar sources (Skals et al. 2003a), as well as their calling behaviour (Acharya and McNeil 1998; Svensson et al. 2003). Moths respond to ultrasound exposure by various manoeuvres, such as diving to the ground, extended zigzagging flight or loops (e.g. Roeder 1964). Standardized ultrasound exposure of moths orienting in odour plumes in the field is almost impossible to achieve, but the controlled environment provided in a flight tunnel offers an excellent system to study decision making by analysing the trade-off between olfactory-guided behaviours and auditory-guided predator avoidance in these insects.

The pyralid moth *Plodia interpunctella* Hübner uses sex pheromones for long-range communication. Females release a blend including four active components, (*Z*,*E*)-9,12-tetradecadienyl acetate (Z9,E12-14:OAc), (*Z*,*E*)-9,12-tetradecadienol (Z9,E12-14:OH), (*Z*)-9-tetradecenyl acetate (Z9-14:OAc) and (*Z*,*E*)-9,12-tetradecadienal (Z9,E12-14:Ald) for attraction of males at large distance (Kuwahara et al. 1971; Zhu et al. 1999). The hearing system in this moth includes a pair of tympanic organs located on the pleuro-ventral side of the first abdominal segment, with four sensory neurons innervating each organ (Mullen and Tsao 1971). These ears are suggested to function both for detection of bat cries and for intra-specific communication (Trematerra and Pavan 1995). Previous studies have shown that long-term ultrasound exposure lower the reproductive success in this species (Svensson et al., 2003; Huang and Subramanyam, 2004).

It was recently shown that male *P*. *interpunctella* orienting in pheromone plumes of optimal quality ignore an ultrasound cue simulating an approaching bat, but show strong evasive reactions to the same acoustic stimulus when orienting towards pheromone sources of less optimal composition or dose (Svensson et al. 2004). Thus, males seem to adjust their predator avoidance behaviour according to the relative quality of the sexual olfactory signal, and similar cross-modal modulation has been observed in the noctuids *Agrotis segetum* Denis & Schiffermüller (Svensson et al. 2004) and *Spodoptera littoralis* Boisduval (Skals et al. 2005). To further investigate the decision-making between reacting to sex odours and ultrasonic stimuli in *P*. *interpunctella*, we here compare the flight behaviour of pheromone-stimulated males when exposed to ultrasound at different intensities. To our knowledge, only one study has so far analysed the flight reactions in pheromone-stimulated male moths when exposed to different intensities of an ultrasound cue. Acharya and McNeil (1998) showed that males of the pyralid *Ostrinia nubilalis* Hübner and the noctuid *Pseudaletia unipuncta* Haworth orienting in sex pheromone plumes reacted stronger to an auditory cue mimicking high predation risk compared to cues signalling moderate or low predation risk. However, the ultrasound signal presented to the moths in that study lasted for 10 s, which is longer than any known bat call, and that may have affected moths in a different way than if a shorter signal had been used.

The aims of the present study were to establish a threshold curve for evasive reactions to ultrasound in *P. interpunctella*, and to investigate both short- and long-term effects of an ultrasound signal on the sexual behaviour in this species. When delivered at increased intensities, the ultrasound cue was expected to trigger (i) stronger immediate evasive reactions in moths as well as (ii) stronger long-lasting effects on their ability to orient in the pheromone plume and reach the odour source.

## Materials and Methods

### Insects

Males of *P. interpunctella* were obtained from a laboratory culture maintained at the Department of Ecology at Lund University for ≈6o generations. This culture was originally established from a laboratory culture at the Danish Pest Infestation Laboratory at Lyngby, Denmark. About 100 males and 100 females from the Central Science Laboratory, Slough, England, were incorporated into the culture after ≈20 generations. Larvae were reared on an artificial diet described by Zhu et al. (1999) at 22 °C and 60% RH. Pupae were separated by sex and placed in a 17:7 L:D photoperiod with the same temperature and humidity as for the larvae. One to five days old males were used in all experiments.

### Chemicals and odour emission system

A sprayer device described by El-Sayed et al. (1999) and later modified according to Skals et al. (2003b) was used for emission of pheromone. The piezoelectric ceramic disc emitted a frequency of 307 kHz, which is far outside the range of frequencies used by echolocating bats and ultrasonic hearing moths, and had no impact on the flight behaviour of males (personal observations). The disc was driven by a Tektronix function generator (CFG 253). As pheromone stimulus, a four-component blend including Z9,E12-14:OAc, Z9,E12-14:OH, Z9-14:OAc and Z9,E12-14:Ald, in the ratios 100/11/18/12, was used (Kuwahara et al. 1971; Zhu et al. 1999). Whereas Z9,E12-14:OAc, Z9,E12-14:OH and Z9-14:OAc were purchased from Bedoukian Research Inc, www.bedoukian.com, Z9,E12-14:Ald was produced from Z9,E12-14:OH in the Department of Chemistry, Royal Institute of Technology, Stockholm, Sweden.

A previous study showed that males flying towards the four-component blend at the optimal emission rate (corresponding to 50 pg min-1 of Z9,E12-14:OAc), did not show strong evasive reactions when exposed to the highest possible intensity produced by our loudspeaker (95 dB SPL) at 50 kHz (Svensson et al. 2004). Therefore, to be able to correlate evasive reactions with sound intensity, we used a lower emission rate (50 fg min-^1^ of Z9,E12-14:OAc), to which moths react more strongly to the ultrasound cue (Svensson et al. 2004).

### Sound emission system

The acoustic stimuli were created by multiplication of signals from a pulse generator (HP 8011A) and a sine wave generator (Wavetek 186) in a custom built trapeze modulator, which added a ramp to the beginning and end of the signal. The rise- and fall time of the ramp was 0.5 ms. The ramp minimizes unwanted spectral artefacts resulting from clicks of the loudspeaker membrane. The signals were attenuated (Kay 865 step attenuator), amplified (Xelex power amplifier) and broadcast through a Technics leaf tweeter (EAS10TH400B). In flight tunnel experiments, each sound stimulus consisted of trains of 10 ms long, ramped pulses at 50 kHz with a repetition rate of 30 pulses s-^1^ and a total stimulus length of 2.5 s. A long stimulus train ensured that moths in flight were exposed to sound in front of the loudspeaker (see also discussion for the reason for stimulus choice). The loudspeaker was positioned 1 m downwind from the sprayer device and at the same height (30 cm) as the pheromone plume and thus the flight path of moths (at 40 cm distance from the loudspeaker). To minimize sound reflections from the opposite wall of the flight tunnel, a 30 cm portion of it was covered with sound absorbing material, which did not disturb the airflow in the middle of the tunnel as checked with TiCl_4_ smoke.

### Estimation of hearing thresholds

To find the frequency of best hearing of male *P. interpunctella*, a threshold curve for the behavioural response to ultrasound (flight stop) was established for tethered moths. Based on this curve, a frequency with lowest threshold for behavioural response could be selected for the sound stimulus used in experiments on free-flying moths (see below). A needle was glued onto the scutum of a moth with wax and the preparation was placed on a holder in the middle of the flight tunnel and at 40 cm distance from the loudspeaker. To monitor wing movements, a custom built infrared emitter-detector was positioned at 5 mm distance and perpendicular to the wing surface of a moth at rest. Wing movements were displayed and recorded on a Tektronix TDS 3012 digital oscilloscope. The preparation was placed in the same flight tunnel as for the experiments with free-flying moths (see below), and moths thus experienced a wind component, which is often essential for initiating and sustaining flight. The moth was forced to initiate flight by touching it with a needle and then stimulated by a sound pulse.

To ensure that the threshold value for the flight-stop response was reproducible and the lowest possible we stimulated with a 50 ms long single pulse. This pulse length corresponds to the integration time for flight-stop response in another pyralid moth (Skals and Surlykke 2000). Flight stop within 300 ms after sound exposure in at least two out of three trials was considered a response. The same individual was tested at frequencies ranging from 20 to 100 kHz in 10 kHz steps, and at intensities ranging from 64 to 98 dB SPL in 4 dB SPL steps. The intensity of a sound stimulus was measured at 40 cm distance from the loudspeaker. The highest sound pressure level not resulting in flight stop according to the criterion above was defined as the hearing threshold for a specific frequency. The latency, i.e., the time from onset of the ultrasound stimulus to flight stop response, was measured and threshold curves were determined for eight males. For each male, frequencies as well as sound pressure levels were randomized to minimize habituation effects. For the same reason, the time span between two sound stimuli was at least 120 s.

### Flight tunnel experiments

Due to the extreme difficulties in standardising the sound-exposure of free-flying moths that are not following a more or less straight path in relation to the loudspeaker, direct comparisons of ultrasound-induced behaviours between pheromone-stimulated and non-stimulated flying moths can only be achieved by using subjects that orient in an odour-plume. The flight responses of pheromone-stimulated male moths when exposed to ultrasound were observed in a 0.9 × 0.9 × 3 m Plexiglas flight tunnel (Valeur and Löfstedt 1996). Flight experiments were conducted one to four hours into the scotophase and the conditions in the flight tunnel were: temperature 21–23 °C, 28–50% RH, wind speed 0.3 m s-^1^ and light intensity 4 lux. Each male was transferred to a small glass cylinder (2.5 cm i.d.) and exposed to the pheromone plume for approximately five seconds and then allowed to take off. Males not leaving the cylinder within three minutes were excluded from the protocol. When a moth was flying in the odour plume at the position of the loudspeaker the ultrasound stimulus was switched on manually. The intensities of the sound cues used were: 0, 77, 83, 89 and 95 dB SPL (measured at 40 cm distance from the loudspeaker).

The behaviour of ultrasound-exposed and unexposed control males was observed for three minutes after they had reached the position of the loudspeaker. Each male was only tested once, and four different behaviours were recorded. Short-term effects of ultrasound exposure, i.e. the proportion of males that dropped to the flight tunnel floor as a response to the sound, as well as the time these males spent immobile on the tunnel floor before resuming flight, were compared at different sound intensities using χ2-statistics and the Kruskal-Wallis nonparametric test followed by a Tukey-Kramer test, respectively. In addition, long-term effects of ultrasound exposure, i.e. the proportion and the time to reach the pheromone source of males after passing the loudspeaker, were compared at different sound intensities. Statistical tests were performed using JMP 3.2.1 (SAS Institute 1997).

## Results

### Hearing threshold curve

**Figure 1.**
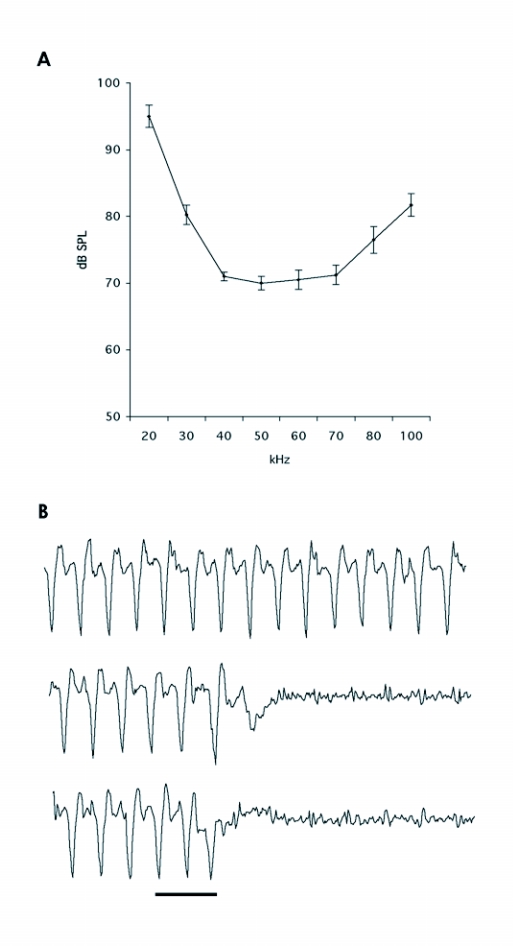
(a) Threshold curve for flight stop of tethered *P. interpunctella* males (n=8). Squares indicate the different intensities of the 50 kHz signal used in flight tunnel experiments, (b) Typical flight stop responses of a male *P. interpunctella* exposed to different intensities of a 50 ms stimulus at 50 kHz. Traces show the wing beat frequency of a male monitored by an infrared detector. The bar indicates the duration of ultrasound stimulation. A flight stop response is observed at both 75 dB SPL and 95 dB SPL, whereas no response is observed at 55 dB SPL, which is below the flight stop threshold of the species. Note that response latency decreases with increasing sound intensity. Intensities of the sound stimulus: upper trace: 55 dB SPL, middle trace: 75 dB SPL, lower trace: 95 dB SPL.

Male *P. interpunctella* had their lowest hearing threshold at frequencies between 40 kHz and 70 kHz. At these frequencies, the flight stop threshold was at ≈70 dB SPL (Figure 1a). Based on the obtained threshold curve, sound stimuli with 50 kHz pulses was used during flight tunnel experiments. The latency time at the frequency with lowest hearing threshold was 71±6 ms, which was very similar to the latency time (72 ms) obtained from another pyralid, *Galleria mellonella* L. (Skals and Surlykke 2000). Figure ib shows typical flight stop responses of a male stimulated with ultrasound at different intensities.

### Flight tunnel experiments

**Figure 2.**
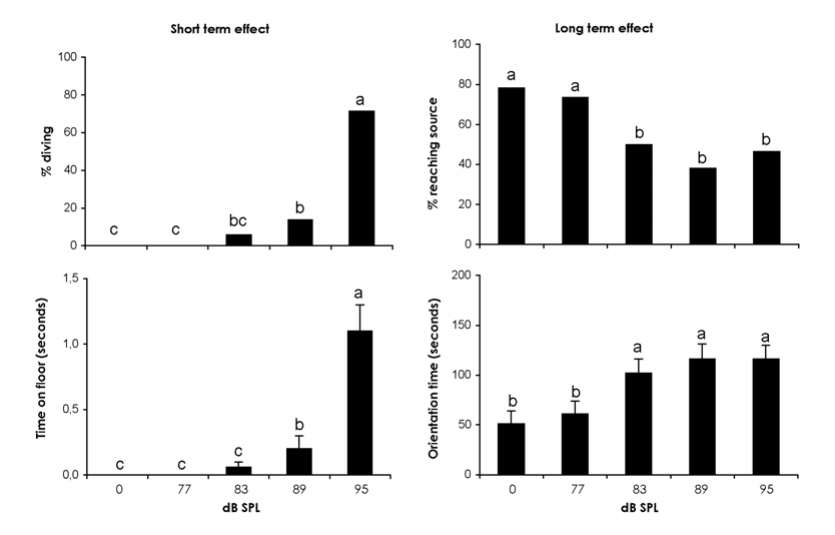
Short- and long-term effects of ultrasound exposure on *P*. *interpunctella* males orienting in a sex pheromone plume. Bars with different letters are statistically different (p < 0.05) according to χ^2^ tests (% diving and % reaching source) and the Kruskal-Wallis nonparametric test followed by a Tukey-Kramer test (time spent on floor and orientation time). The intensities of the auditory cue used for the different groups are shown below the graphs. Between 28 and 34 males were tested per group.

No evasive behaviours could be distinguished in any orienting male when exposed to no ultrasound or ultrasound at 77 dB SPL. At 83 dB SPL, two males out of 34 reacted to the sound by cessation of flight and diving to the flight tunnel floor (Figure 2). Other males showed small evasive reactions by extending zigzagging flight. At 89 dB SPL, four males out of 29 dived to the flight tunnel floor after sound exposure and others showed extending zigzagging, loops etc. At 95 dB SPL, the majority (20 out of 28) reacted to the sound by diving. Intensities of ≥89 dB SPL were needed to induce significantly more diving responses in ultrasound-exposed males compared to unexposed ones. In contrast, the time spent on the flight tunnel floor as a response to the ultrasound cue was only significantly different from zero for males exposed to ultrasound at 95 dB SPL (Figure 2). Males exposed to 95 dB SPL spent on average five times longer time on the floor compared to those exposed to 89 dB SPL.

Seventy-eight percent of the control moths that were not exposed to any ultrasound reached the odour source within the time limit after they had passed the loudspeaker (Figure 2). The ability of males to relocate the plume and orient to the pheromone source after ultrasound stimulation depended on the intensity of the signal. No difference in proportions of moths reaching the odour source was observed between control males and those exposed to 77 dB SPL. However, significantly fewer ultrasound-exposed males reached the odour source compared to unexposed males when stimulated by ultrasound at ≥83 dB SPL. Also, males exposed to ultrasound at ≥83 dB SPL took significantly longer time to reach the pheromone source (Figure 2), and this effect was independent of whether the orientation time for ultrasound-exposed males that dropped to the floor was measured from the time they were exposed to the sound cue or from the time they resumed flight after sitting on the flight tunnel floor.

## Discussion

The ultrasound pulses clearly affected the pheromone-mediated flight behaviour of male *P*. *interpunctella*, but only at the highest sound pressure levels. This suggests that males engaged in upwind flight towards a calling female adjust their sound-induced manoeuvres according to the strength of the signal. These results, in combination with the earlier observations that males adjust their evasive behaviours to ultrasound depending on the quality of the pheromone signal (Svensson et al. 2004), reveal a complex and dynamic integration of the conflicting sensory stimuli perceived by the moth. Skals et al. (2005) did a fine-grained analysis of this cross-modal modulation in *S. littoralis* by simultaneously exposing walking males to pheromone and ultrasound stimuli at different relative strengths. The threshold for evasive behaviour (freezing) increased by 40 dB SPL in males stimulated by a female extract (i.e. the complete sex pheromone blend) compared to those not stimulated by any odour. In a natural situation, such reduction in the evasive response in pheromone-stimulated males will make them more vulnerable to an attacking bat, because they will evade the predator at a later stage, hence trading reproduction with a greater risk of predation.

Both short- and long-term effects of ultrasound exposure were observed. When stimulated with 95 dB SPL pulses, significantly more males dropped to the flight tunnel floor and spent longer time in a freezing posture on the floor compared to males exposed to ultrasound at lower intensities. Interestingly, following stimulation by higher sound intensities, males had problems relocating and locking on to the pheromone plume after loosing it as a consequence of dropping to the flight tunnel floor, as compared to males exposed to lower sound intensities.

Although such males at a later stage flew through the odour plume, some of them did not resume upwind flight. Some orienting males that showed small or no visible reactions upon ultrasound stimulation were unable to continue their flight to the source, but instead showed arrestment behaviour and then left the pheromone plume. Fewer moths reached the odour source following exposure to high sound intensities (83–95 dB SPL) compared to lower intensities (77 dB SPL or no sound), and those moths that reached the odour source spent more time on the orientation flight. This indicates that either the sound or the evasive reaction *per se* disrupted the pheromone search behaviour to an extent where these moths had a significant disadvantage in reaching the pheromone source compared with moths that flew without sound stimulation. It is premature to speculate about how the olfactory machinery at a higher neural level is affected by the ultrasound cue even several minutes after the exposure event, but it brings up interesting questions on how animal decision-making must be based on cross-modal perception.

Tethered males had their lowest flight stop threshold at frequencies between 40 kHz and 70 kHz (Figure 1a), corresponding to the frequencies used by many echolocating bats (Popper and Fay 1995; Parsons and Jones 2000). This is similar to two other pyralid species, *Ephestia kuehniella* Zeller and G. *mellonella*, both with best hearing at 60 kHz (Pérez and Zhantiev 1976; Skals and Surlykke 2000). At 50 kHz, the threshold for flight stop in *P. interpunctella* was ≈70 dB SPL. In addition, the latency times of *P*. *interpunctella* and *G*. *mellonella* to ultrasound were very similar, suggesting that the hearing system in pyralid moths may show little inter-specific variation. Skals and Surlykke (2000) estimated the flight stop threshold of *G. mellonella* at 60 kHz to 72 dB SPL, which was 25 dB above the sensory threshold, based on electrophysiologically determined audiograms. If the same difference exists between sensory and behavioural thresholds in *P*. *interpunctella*, its sensory threshold at frequencies of best hearing should be ≈45 dB SPL. In the flight tunnel we noticed that a majority of moths dived when they were exposed to a sound pressure level of 95 dB. If the sensory threshold is as low as 45 dB SPL it means that the moths may have additional graded responses, which are elicited in the range from 45 to 95 dB SPL. These low intensity elicited responses may involve steering away from the sound source or similar less obvious behavioural responses. Figure 2 indicates that this may happen, since moths stimulated by 83 dB SPL on average took as long time to reach the odour source as those stimulated by 95 dB SPL.

Echolocation signals used by bats for orientation and prey detection vary considerably among species (Fenton et al. 1980; Fenton 1995; Neuweiler 1990; Water and Jones 1995; Jones 1999). With such great variation observed in call design among bat species, and the various bat predators *P*. *interpunctella* is potentially exposed to, these insects must be able to process sound stimuli that differ in pulse length, pulse repetition rate, and duty cycle, for efficient predator avoidance. The sound stimuli were not constructed to imitate a particular bat species, but to apply a signal (with all parameters well within the range documented in bat calls in nature), which elicited consistent behavioural responses to ultrasound. This was important because we were comparing the signal strength between acoustic and odour stimuli. A natural bat cry consists of a search, approach and a terminal phase, which are characterised by different content of acoustic energy. If a “natural” bat cry had been used, one could not be sure if the moths responded to the high-energy phase (terminal phase) in the stimulus or the low-energy phase (search phase), and therefore the results could not be compared between individuals. A similar approach has been used in previous publications on bat-moth interactions and integration of conflicting bimodal information (Acharya and McNeil 1998; Skals and Surlykke 2000; Jones et al. 2002; Skals et al. 2003; 2005; Svensson et al. 2004). Stimulating the moth by 95 dB SPL corresponds to a bat cry at 1–2 m distance from the target.

To summarize, the results obtained in this study add to previous findings that the trade-off between odour-induced attraction and sound-induced avoidance in male moths is dynamic and depends on the relative strengths of the conflicting olfactory and auditory stimuli perceived. Multi-modal approaches during investigations of sensory-guided behaviours in insects should be encouraged, as these organisms live in complex environments and have to continuously integrate signals detected by their different sensory systems. Ultrasonic hearing moths with a broad repertoire of odour-guided behaviours offer unique opportunities to study how insects adjust important behaviours, such as mating, oviposition and foraging, in relation to the risk of predation. Therefore, we believe that this bimodal system can develop into an important model to answer questions within the field of cognitive ecology. Future studies will focus on whether those males that quickly relocated the pheromone source after a perfect acoustic defence are super-males, i.e. do they have a reproductive advantage besides coming first?
